# Delivering the diagnosis of multiple system atrophy: a multicenter survey on Japanese neurologists’ perspectives

**DOI:** 10.1186/s12883-024-03666-4

**Published:** 2024-05-13

**Authors:** Miki Yoshitake, Atsuhiko Sugiyama, Takayoshi Shimohata, Nobuyuki Araki, Masahide Suzuki, Kazumoto Shibuya, Kengo Nagashima, Nobutaka Hattori, Satoshi Kuwabara

**Affiliations:** 1 Department of Neurology, Graduate School of Medicine, Chiba University, 1-8-1 Inohana, Chuo-Ku, Chiba, 260-8677 Japan; 2 Department of Neurology, Juntendo University Hospital, Tokyo, Japan; 3 Department of Neurology, Gifu University Graduate School of Medicine, Gifu, Japan; 4 Department of Community-Oriented Medical Education, Chiba University Graduate School of Medicine, Chiba, Japan; 5 Department of Neurology, Chiba Rosai Hospital, Chiba, Japan; 6 Biostatistics Unit, Clinical and Translational Research Center, Keio University Hospital, Tokyo, Japan

**Keywords:** Critical incident technique, Differential diagnosis, Neurologists, Sudden death, Multiple system atrophy, Breaking the diagnosis

## Abstract

**Background:**

Multiple system atrophy (MSA) is a progressive, incurable, life-threatening neurodegenerative disease uniquely characterized by the risk of sudden death, which makes diagnosis delivery challenging for neurologists. Empirical studies on breaking a diagnosis of MSA are scarce, with no guidelines currently established. This study aimed to investigate neurologists’ current practices and experiences in delivering the diagnosis of MSA.

**Methods:**

We conducted a multicenter online survey and employed a mixed-methods (quantitative and qualitative) study design in which responses to open-ended questions were analyzed qualitatively using critical incident technique.

**Results:**

Among the 194 neurologists surveyed, 166 opened the survey (response rate = 85.6%), of whom 144 respondents across various Japanese regions completed the survey. Accordingly, 92.3% and 82.8% of the participating neurologists perceived delivering the diagnosis of MSA and explaining the risk of sudden death as difficult, respectively. Factors independently associated with difficulties in diagnosis delivery included explaining the importance of the family decision making process in life-prolonging treatment, perceived difficulties in delivering information regarding the risk of sudden death, and perceived difficulties in differential diagnosis of MSA.

**Conclusions:**

Our findings showed that the majority of neurologists perceived delivering the diagnosis of MSA and explaining the risk of sudden death as difficult, which could have been associated with the difficulty of breaking the diagnosis of MSA. Difficulty in conveying bad news in MSA are caused by various factors, such as empathic burden on neurologists caused by the progressive and incurable nature of MSA, the need to explain complex and important details, including the importance of the family decision-making process in life-prolonging treatment, difficulty of MSA diagnosis, and communication barriers posed by mental status and cognitive impairment in patients or their family members. Neurologists consider various factors in explaining the risk of sudden death (e.g., patient’s personality, mental state, and degree of acceptance and understanding) and adjust their manner of communication, such as limiting their communication on such matters or avoiding the use of the term “sudden death” in the early stages of the disease. Although neurologists endeavor to meet the basic standards of good practice, there is room for the multiple aspects for improvement.

**Supplementary Information:**

The online version contains supplementary material available at 10.1186/s12883-024-03666-4.

## Background

Multiple system atrophy (MSA) is an adult-onset, rapidly progressive, and pontifically fatal neurodegenerative disease characterized by autonomic failure, poorly L-Dopa-responsive parkinsonism, cerebellar ataxia, pyramidal signs, or any combination of the mentioned characteristics [1]. Given its variable clinical presentation, diagnosing MSA throughout its disease course is quite challenging. Indeed, recent clinicopathological studies have shown an overall suboptimal diagnostic accuracy and sensitivity, especially at the early disease stages [2–4]. Moreover, pharmacological treatments that could cure MSA or halt its progression are currently still lacking [5]. MSA progresses faster than Parkinson’s disease or spinocerebellar ataxia despite having similar symptoms and sharing a degenerative nature [6–8]. Upon initial clinical presentation, patients with MSA have demonstrated a mean survival of 6–10 years [1].

Given the difficulty in diagnosing MSA in its early stages, the lack of disease-modifying therapy, and the rapid disease progression, delivering the diagnosis of MSA to patients and their families is expectedly challenging for neurologists. Indeed, studies show that neurologists experience difficulty and emotional burden when delivering the diagnosis of motor neuron disease, which shares its progressive, incurable, and life-threatening characteristics with MSA [9, 10]. Moreover, vocal cord abductor paralysis, abnormal breathing control, and cardiac autonomic dysfunction among patients with MSA place them are at risk of sudden death, which is difficult to fully predict or prevent [11]. Vocal cord paralysis and abnormal breathing, which can cause sudden death, can occur even at the early stages of the disease [12, 13]. This distinctive feature of sudden death in MSA makes diagnosis delivery even more difficult for neurologists, intensifying the conflict between accountability and empathic distress. However, empirical research on delivering the diagnosis of MSA from the neurologist's perspective is scarce, with no guidelines having been established to date.

The current study aimed to (1) comprehensively survey the current practices and experiences in delivering the diagnosis of MSA from the neurologists’ perspectives and identify key clinical issues that would aid in the development of clinical guidelines for breaking the diagnosis of MSA and (2) determine the extent of difficulty in delivering the diagnosis of MSA and factors affecting it.

## Methods

### Design

A cross-sectional mixed-methods (quantitative and qualitative) study design was used.

### Ethical considerations

This study was approved by the Institutional Review Board of the Chiba University Graduate School of Medicine. All participants were informed that participation was voluntary. Completing the survey implied consent for study participation. The first page of the survey contained comprehensive information regarding the study, including an explanation that this was a questionnaire survey about delivering the diagnosis of MSA and that “delivering the diagnosis” is defined as “informing the patient and their family about the diagnosis and disease prognosis, as well as treatment possibilities and limitations.”

### Survey

The questionnaire used was constructed based largely on the relevant literature on breaking bad news and guidelines such as the SPIKES protocol and the European Federation of Neurological Societies (EFNS) guidelines on breaking the news of amyotrophic lateral sclerosis (ALS) [14, 15]. The first draft of the survey was reviewed for clarity and relevance by two board-certified neurologists (A.S. and T.S.), with adjustments being made based on their comments. The survey comprised 51 questions grouped into three parts. Part 1 included closed questions about demographics and clinical experiences in MSA. Part 2 consisted of questions about current practices and difficulties in delivering the diagnosis of MSA rated on a 5-point Likert scale. Part 3 comprised three open-ended questions asking about personal experiences in delivering the diagnosis of MSA. The survey was conducted online between July and August 2022 using the Qualtrics platform (Qualtrics LLC, Provo, UT). Participants were recruited through the Research Committee for Ataxic Disease and the Movement Disorders Society of Japan, excluding those without experience in delivering the diagnosis of MSA.

### Qualitative analysis

The critical incident technique, an inductive qualitative analysis method developed by Flanagan [16], has been utilized to measure typical performance and create operating procedures and tasks across various disciplines [17]. We used this technique to comprehensively determine the elements that made delivering the diagnosis of MSA difficult from the participants’ perspective. First, through the process of discussion and consensus between a clinical psychologist (M.Y.) and a board-certified neurologist (A.S.) (categorizing team), critical incidents from open-ended answers were extracted. Similar incidents were then merged into a category, and category labels were determined preliminarily. Second, an external reviewer (T.S.) validated the grouping categories and fitness between each category and label. Through consensus between the categorizing team and external reviewer, the final category set was established. Third, to ensure accuracy and reduce bias, two other board-certified neurologists (N.A. and M.S. in the sorting team) independently sorted each critical incident into the final category set. Thereafter, Cohen’s κ for the sorting team was calculated. Finally, frequency analysis was conducted to determine the number and percentage of each category.

### Statistical analysis

Spearman’s coefficient and Kendall’s correlation coefficient were used to determine the relationship between difficulties in diagnosis delivery (ordinal variable) and continuous variables and between difficulties in diagnosis delivery and ordinal variables. Variables significantly correlated with difficulties in diagnosis delivery were included in linear regression analyses to identify factors independently associated with difficulties in diagnosis delivery. All statistical analyses were performed using SPSS software version 25.0 (SPSS Japan, Tokyo, Japan), with a two-sided *p* value of < 0.05 indicating statistical significance.

## Results

### Response rate and respondent profile

Among the 194 neurologists surveyed, 166 opened the survey (response rate = 85.6%). Of the 166 who opened the survey, 12 and 10 were excluded for having no experience in delivering the diagnosis of MSA and for not completing the survey. Thus, 144 respondents (74.2%) were ultimately included for analysis. This number represents around one-sixth of the physicians belonging to the Movement Disorders Society of Japan (for reference, approximately 9,000 neurologists are currently practicing in Japan). Table 1 summarizes the characteristics of the respondents. All 144 respondents answered all questions except for Q46. Except those who answered “never” in Q45, all respondents answered Q46, as indicated in the survey instructions.

**Table 1:** Respondent characteristics

Sex, n (%)	
Female,	26 (18.1)
Male	116 (80.6)
Prefer not to answer	2 (1.4)
Missing	0 (0)
Age, median (IQR)	48 (36.5–59.5)
Age, n (%)	
30–39	42 (29.2)
40–49	35 (24.3)
50–59	21 (14.6)
60–69	27 (18.8)
70 or older	19 (13.2)
Missing	0 (0)
Years in practice of delivering the diagnosis of MSA, n (%)	
0–9	49 (34.0)
10–19	50 (34.7)
20–29	33 (22.9)
30–39	12 (8.3)
Missing	0 (0)
Times the diagnosis MSA was delivered per year, n (%)	
< 1	13 (9.0)
1–4	103 (71.5)
5–9	16 (11.1)
10–14	7 (4.9)
15 or more	5 (3.5)
Missing	0 (0)
Region, n (%)	
Hokkaido	1 (0.7)
Tohoku	7 (4.9)
Kanto	77 (53.5)
Chubu	30 (20.8)
Kansai/Kinki	13 (9.0)
Chugoku	3 (2.1)
Shikoku	7 (4.9)
Kyushu/Okinawa	6 (4.2)
Missing	0 (0)

*IQR* inter-quartile range, *MSA* multiple system atrophy

### Difficulties in delivering the diagnosis of MSA

**Fig. 1:**
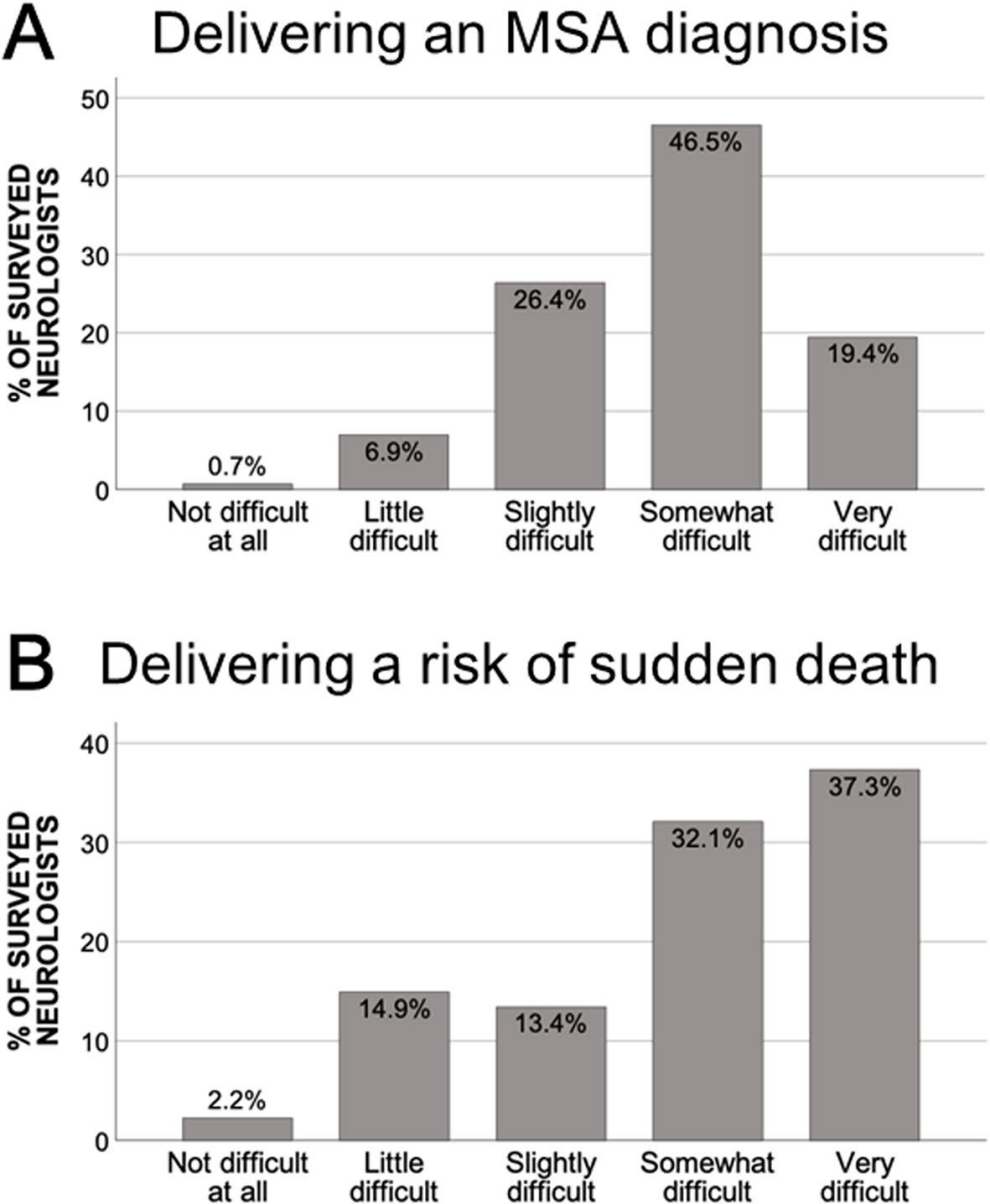
Difficulty levels in delivering the diagnosis of multiple system atrophy (MSA)

Over 90% (92.3%) of the participating neurologists found delivering the diagnosis of MSA to be “very to somewhat difficult,” whereas over 80% (82.8%) found explaining the risk of sudden death to be “very to somewhat difficult” (Fig. 1). Over 70% of the participants reported “always” or “usually” providing an explanation to the patients and their families regarding the risk for sudden death (75.7%) and its causes (70.9%). Around 70% of the participants stated they adjust their decision to communicate regarding the risk of sudden death based on the patients’ disease stage. Moreover, 26.4% of the participants reported that they “occasionally” get emotionally upset while delivering the diagnosis of MSA, whereas 11.1% reported never experiencing such feelings.

To investigate factors associated with neurologists-perceived difficulties in delivering the diagnosis of MSA, univariate correlation analysis was performed (Supplementary Table 1). Difficulties in delivering the diagnosis of MSA were significantly correlated with neurologist age (*ρ* =  − 0.23, *p* = 0.005), years in practice of delivering the diagnosis of MSA (*ρ* =  − 0.2, *p* = 0.015), number of times the diagnosis of MSA was delivered per year (*τ* =  − 0.17, *p* = 0.019), preparing the details to be communicated before delivering the diagnosis of MSA (*τ* = 0.26,* p* < 0.001), ascertaining the patient’s understanding of MSA (*τ* = 0.14, *p* = 0.048), explaining the importance of the family decision making process in life-prolonging treatment (*τ* = 0.15, *p* = 0.046), asking the patient’s support network to be present (relatives) (*τ* = 0.19, *p* = 0.015), getting emotionally upset when delivering the diagnosis of MSA (*τ* = 0.21, *p* = 0.005), perceived difficulties in explaining the risk of sudden death (*τ* = 0.36, *p* < 0.001), and perceived difficulties in the differential diagnosis of MSA (*τ* = 0.38, *p* < 0.001).

Multiple linear regression analysis (Table 2) found that explaining the importance of the family decision making process in life-prolonging treatment (*p* = 0.023), perceived difficulties in delivering a risk of sudden death (*p* < 0.001), and perceived difficulties in differential diagnosis of MSA (*p* < 0.001) were independently associated with difficulties in diagnosis delivery.

**Table 2:** Factors associated with difficulties in delivering the diagnosis of MSA using linear multiple regression analyses

Variables				
	Regression coefficient estimate	95% CI		*p* value
Characteristics of neurologists				
Age	− 0.003	− 0.014	0.008	0.589
Years in practice of delivering the diagnosis of MSA	< 0.001	− 0.018	0.018	0.992
Number of delivering the diagnosis of MSA per year	− 0.117	− 0.249	0.015	0.081
Preparation for delivering the diagnosis of MSA				
Preparing the details to be communicated before delivering the diagnosis of MSA	0.054	− 0.114	0.222	0.525
Ascertaining the patient’s understanding of MSA	0.028	− 0.081	0.136	0.618
Providing information when delivering the diagnosis of MSA				
Explaining the importance of the family decision making process on life-prolonging treatment	0.163	0.023	0.303	**0.023**
Structure when delivering the diagnosis of MSA				
Asking the patient’s support network when present (relatives)	0.055	− 0.165	0.275	0.621
Communication when delivering the diagnosis of MSA				
Neurologist's emotional upsetting when delivering the diagnosis of MSA	0.148	− 0.015	0.311	0.075
Magnitude of difficulties in delivering the diagnosis of MSA				
Perceived difficulties in delivering a risk of sudden death	0.252	0.131	0.374	**< 0.001**
Perceived difficulties in differential diagnosis of MSA	0.341	0.197	0.485	**< 0.001**

*MSA* multiple system atrophy, *SE* standard error

### Current practices in delivering the diagnosis of MSA

All questions and responses in the survey are depicted in Supplementary Table 2. When asked about the setting of the consultation, 73.3% of the participants reported “always” or “usually” delivering the diagnosis in a private space, while several reported “rarely” or “never” delivering the diagnosis in a private space. Almost all (95.1%) stated “always” or “usually” asking close relatives of the patients to be present during the consultation. Almost all (93.0%) reported “always” or “usually” tailoring the details to be communicated to suit the patient’s medical condition. Around 80% reported that they “always” or “usually” prepared the details to be communicated to the patient and their families in advance and ascertain the patient’s understanding beforehand. However, only around half stated that they “always” or “usually” ascertained the extent of the information wanted by the patient and informed the patient that they had some bad news before delivering the diagnosis of MSA. Over 70% performed two or more consultations, but over half reported that each consultation lasted < 30 min. Moreover, 22.9% reported “never” asking relevant co-medicals to be present during diagnosis delivery.

When asked about communication manners during diagnosis delivery, almost all participants reported “always” or “usually” talking at the patient's pace, making an effort to explain using simple and straightforward terminology, approaching the patient sympathetically, making eye contact with the patient, and accepting the emotions the patient expresses.

In terms of providing information about MSA, almost all participants reported “always” or “usually” informing the patient that no radical treatment is available, whereas < 50% reported “always” informing the patient regarding the average years of survival. Approximately 50%–60% reported “always” or “usually” informing the patient that, on average, they will be wheelchair-bound within around 5 years after onset, that they will be bedridden within 7–8 years of onset, that they may lose the ability to speak, and that they may need self-catheterization or bladder catheterization. However, only around 30% reported “always” or “usually” informing the patient about the possibility of accompanying cognitive dysfunction. Moreover, 68.1%, 63.9%, and 51.4% reported “always” or “usually” informing that gastrostomy, tracheostomy, or invasive positive pressure ventilation using an artificial respirator may be needed or used, respectively. Around four-thirds of the participants stated “always” or “usually” informing the patient regarding the importance of talking about life-prolonging treatments in advance with their families.

To reassure their patients, over 90% of the participants stated “always” or “usually” informing the patients that rehabilitation is an option, complications can be alleviated with symptomatic treatment, they will not be abandoned, and their treatment will be continued. Over half of the participants reported “always” or “usually” informing the patient that a second opinion can be received when needed. Furthermore, 36.1% “always” or “usually” shared information regarding patient support groups.

### Qualitative analysis of open-ended questions on personal experience in delivering the diagnosis of MSA

A total of 40 categories were created from three open-ended questions. The interrater reliability among the sorting team (N.A. and M.S.) was κ = 0.991, 0.684, and 0.768 for Q1, Q2, and Q3, respectively, indicating “very good” to “excellent” agreement. Table 3 lists all major categories (> 10% in frequency) that emerged from each question, whereas Supplementary Table 3 summarizes all categories and representative quotations. We present each question, the number of responses to each, the number of categories that emerged from the responses, and the characteristics of the categories that emerged below, along with quotes from representative responses.

**Table 3:** Major categories (> 10% in frequency) that emerged after analyzing the free descriptive answers

Q1. Please describe freely the situations in which you felt that delivering the diagnosis of MSA was particularly difficult	
Categories	No. (%)
Having to give bad news about the prognosis and treatment	20 (16.3)
Being in a situation where the patient does not understand	18 (14.6)
Being in a situation where tailored information is needed according to the patient’s symptoms and level of progression	15 (12.2)
Q2. With regard to explaining sudden death when delivering the diagnosis of MSA, on what basis do you sense difficulty?	
Categories	No. (%)
Considering the mental damage faced by the patient leading to hesitation in telling the truth	21 (18.4)
Understanding the disclosure timing	13 (11.4)
Lack of established treatment to prevent sudden death	13 (11.4)
Understanding patient acceptance of the risk of sudden death	12 (10.5)
Q3. How do you tailor your explanation about the risk of sudden death?	
Categories	No. (%)
Avoiding proactive explanation regarding the details on the risk of sudden death at an early stage	33 (25.4)
Communicating in a gradual manner	26 (20.0)
Considering the patient's level of acceptance and understanding of the disease	20 (15.4)
Considering the degree to which the patient is at risk of sudden death	18 (13.8)

*MSA* multiple system atrophy

#### Q1. “Please describe freely the situations in which you felt that delivering the diagnosis of MSA was particularly difficult.”

From the 99 responses to Q1, 123 critical incidents were extracted and 15 categories were created. The most common difficulty faced during diagnosis delivery was breaking the bad news about prognosis and treatment (20, 16.3%). Other difficulties included communicating the diagnosis to young patients and the conflict between the responsibility to explain and empathic pain. These difficulties represent the physician’s psychological burden when delivering devastating information regarding MSA to patients and their families. One respondent noted that:

*“I feel distressed by the large amount of bad news, such as the fact that there is no effective treatment, the patient would become bedridden, and there is a risk of sudden death.”*

Diagnostic uncertainty and the complexity of the symptoms and clinical course of MSA also made diagnosis delivery difficult. Given the complexity of MSA, the difficulty in ensuring that the patients and their families gained understanding and the need to tailor the explanation to the patient’s symptoms and level of progression further increased the difficulty of diagnosis delivery. Apart from explaining the complex symptoms and clinical course of MSA, the need to make decisions on important matters, such as gastrostomy and ventilator use, also contributed to the difficulties:

*“Disclosing at a stage when the diagnosis of MSA is suspected.”*

*“Sometimes I need to change the details of the explanation to suit the level of disease progression.”*

Factors on the receiving end, such as patient and family personality, acceptance, lack of family support, excessive expectations, being overly optimistic, and presence of a mental illness, also complicated diagnosis delivery.

#### Q2. “With regard to explaining sudden death when delivering the diagnosis of MSA, on what basis do you sense difficulty?”

From the 99 responses to Q2, 114 critical incidents were extracted and 14 categories were created. The most frequent reason for the difficulty in explaining the risk of sudden death was the physician's empathic burden, which made them hesitant to break the truth given the psychological shock they expected the patient to receive (21, 18.4%). One respondent noted the following:

*“I feel reluctant to tell the truth when I imagine the shock that the patient will receive.”*

The lack of information regarding the characteristics of the patients receiving information on sudden death and the extent of information on sudden death needed to be shared also increased the neurologist's empathic burden:

*“Because it is a shocking fact, I am careful as to how I communicate the details.”*

The lack of predictability, lack of established treatment or preventive methods, diverse causes, infrequent yet serious events, and the need to ensure the patient's understanding and acceptance and ascertain the degree to which patients understood and accepted also contributed to difficulties in explaining sudden death.

#### Q3. “How do you tailor your explanation about the risk of sudden death?”

From the 103 responses to Q3, 130 critical incidents were extracted and 11 categories were created. The most frequent method by which neurologists adjusted their explanation on the risk of sudden death was to avoid proactively providing details on sudden death at the early stages of the disease (33, 25.4%). One respondent noted that:

*“At an early stage, I do not explain the details of sudden death unless asked by the patient and his/her family.”*

Aside from disease stage, the patient's personality, mental state, acceptance, understanding, risk of sudden death, and family relationships were considered when explaining the risk of sudden death. Hence, the risk of sudden death was occasionally disclosed only to family members:

*“Depending on the circumstances, I inform the family first.”*

Communication manners were adjusted to dampen the impact of information regarding sudden death risk. This involved communicating gradually or explaining that sudden death is less frequent at the early stages and avoiding the term “sudden death” at the early stages.

## Discussion

This has been the first multicenter survey in Japan on delivering the diagnosis of MSA from the neurologists’ perspective. Notably, the high response rate (85.6%) obtained from neurologists across Japan and subsequent quantitative analysis of the data provided comprehensive insight into current practices and experiences surrounding the diagnosis of MSA. Qualitative analysis of open-ended questions using the critical incident technique enhanced the understanding of the quantitative results.

Participating neurologists perceived delivering the diagnosis of MSA as a challenging yet crucial aspect of their role, with over 90% finding diagnosis delivery difficult. The magnitude of difficulty observed herein was comparable to or higher than those previously reported when delivering the diagnosis of ALS (69.1%) [9] or motor neurodegenerative diseases (77%) [10]. The current study found that difficulties in the differential diagnosis of MSA and explaining the importance of family decision making process in life-prolonging treatment were independently associated with neurologist-perceived difficulties in diagnosis delivery. Consistent with these quantitative findings, qualitative analysis revealed that the uncertainty of MSA diagnosis and the need to make decisions on important matters, such as gastrostomy and ventilator use, contributed to the difficulties. Previous reports have noted that physicians find end-of-life discussions, including life-prolonging treatment, challenging due to fear of legal consequences, ambiguity in patient outcome goals, and limited knowledge and skills in end-of-life care [18, 19]. Difficulties in end-of-life discussions in neurodegenerative diseases, such as Parkinson's disease and ALS, have also been noted, including timing and the need for sufficient time [20, 21].

Neurologists perceived explaining the risk of sudden death as challenging, which might be associated with the difficulty of delivering the diagnosis of MSA. In fact, over 80% of neurologists participating in the current study found explaining the risk of sudden death difficult. Multiple regression analysis revealed that difficulty in explaining sudden death risk was independently associated with difficulty in delivering the diagnosis of MSA. Sudden death in MSA, which is difficult to fully predict or prevent, has been attributed to suffocation resulting from vocal cord abductor paralysis, abnormal control of breathing, and cardiac autonomic dysfunction [11]. Although vocal cord paralysis and abnormal breathing can occur early in the disease [12, 13], emphasizing concerns about sudden death risk at the early disease stages may adversely impact patients. Therefore, clinical ethical issues regarding when and how neurologists should inform patients regarding the risk of sudden death have been raised [11]. Indeed, our qualitative analysis indicated that the diverse causes and timing of sudden death, lack of established prevention methods, and uncertainty of optimal timing contributed to the difficulties in explaining the risk of sudden death. We found that neurologists consider various conditions and adjust their manner of communication to lessen the impact of information regarding the risk of sudden death. Specifically, they considered the patient’s personality, mental state, degree of acceptance and understanding, and actual risk of sudden death. To damp the impact of information regarding the risk of sudden death, neurologists adjusted their communication such that they limited their communication on sudden death risk and avoided using the term “sudden death” at the early stages.

Besides the direct association between the difficulty in explaining the risk of sudden death and the difficulty in breaking MSA diagnosis, background factors common to both difficulties could have played a role in detecting this association. Qualitative analysis revealed that empathic distress due to revealing shocking truths to the patients or their families was a common contributor to both difficulties. Over 25% of our participants reported occasionally experiencing emotional distress while delivering the diagnosis of MSA. Consistent with our results, previous studies have also revealed that physicians find it emotionally burdening and stressful to break bad news to patients with motor neurodegenerative diseases [10, 22]. Communication barriers due to mental status and cognitive impairment caused problems in acceptance and understanding and contributed to difficulties in both explaining sudden death risk and delivering the diagnosis of MSA. Although cognitive impairment has traditionally been believed to rarely develop in MSA, recent evidence has indicated that cognitive impairment is an integral part of MSA [23]. Studies have also shown that some MSA patients develop psychiatric symptoms, including depression and anxiety [23]. Although one study estimated that clinically significant cognitive symptoms appear an average of 7 years after diagnosis [24], some cases with early cognitive impairment have been reported [25, 26]. To support patient autonomy in making decisions regarding important treatment options, such as gastrostomy, tracheostomy, and invasive positive pressure ventilation using an artificial respirator, assessing cognitive function and adjusting the manner and timing of MSA diagnosis delivery to account for cognitive function may be important. Conversely, more than a third of our participants reported never or rarely communicating about the possibility of accompanying cognitive dysfunction, which could be an area for improvement.

Generally, participants seemed to satisfy communication manner-related standards of good practice [14] when breaking bad news by talking at the patient’s pace, endeavoring to explain using simple and straightforward terminology, approaching the patient sympathetically, making eye contact, and accepting the emotions they express. Nonetheless, we found that participants could improve the setting at which they delivered the diagnosis of MSA. Certain participants (15.9%) “rarely” or “never” delivered the diagnosis in a quiet place where no interruptions would occur and failed to satisfy the general recommendations for breaking bad news [14]. Although over 70% of the participants reported delivering the diagnosis gradually, over half reported that each consultation took < 30 min. The EFNS guidelines on the clinical management of ALS recommend delivering the diagnosis of ALS within 45–60 min [15]. A previous survey on breaking the diagnosis of ALS from the patient’s perspective showed that those who spent more time with their neurologists had a better perception of the neurologists’ abilities/skills and were more satisfied with the delivery process [27]. Given that MSA, like ALS, is a progressive, incurable, and life-threatening disease, with the added unique feature of sudden death, the consultation time in current practice is likely to be short. Although the current study found no significant correlation between consultation time and difficulties perceived by neurologists, a survey from the patient’s perspective may reveal a relationship between both factors. Only a little over 50% of our participants always or usually ascertained how much the patient wanted to know about MSA and provided a warning statement on the upcoming bad news. Obtaining the patient’s invitation by ascertaining how much the patient wants to know has been considered one of the steps in breaking bad news [14]. Studies have shown that warning the patient regarding the upcoming bad news, a strategy called “forecasting,” may lessen the shock following the breaking of bad news and may facilitate information processing [28, 29].

We also found that neurologists currently engaged in clinical practice could improve on reassurance. Only about a third of our participants reported “always” or “usually” providing information about patient support groups. Approximately 20% of the participants reported “rarely” or “never” informing patients about getting a second opinion if needed. The EFNS guidelines on the clinical management of ALS recommend that neurologists provide information on patient support groups and acknowledge a willingness to get a second opinion if the patient wishes [15].

The current study has several limitations worth noting. First, selection bias may have been present given that most neurologists who completed the survey were most likely interested in the topic. Moreover, a majority of participants were neurologists who specialized in movement disorders probably owing to our recruitment through the Research Committee for Ataxic Disease and the Movement Disorders Society of Japan. Therefore, the results of this survey might not reflect all neurologists. Second, this was a national survey throughout Japan. Considering the diversity in medical ethics laws or views on life and death across different countries, the findings of this study may not be applicable to neurologists in countries other than Japan. Third, this survey did not ask neurologists whether they applied diagnostic criteria to establish a diagnosis of MSA or at what level of diagnostic certainty they usually deliver the diagnosis, which may differ between movement disorder specialists and non-movement disorder specialists. Furthermore, neurologists’ behavior and the difficulties they perceive when delivering the diagnosis may vary depending on the certainty of the diagnosis. Further research is needed to clarify these points, including questions regarding whether or not to apply diagnostic criteria and at what point of diagnostic certainty based on the diagnostic criteria should the diagnosis be delivered. Finally, not all respondents included in this survey were experts in MSA practice, with around one-third having 0–9 years of experience in delivering the diagnosis of MSA. This may have been due to our failure to specify the number of years of experience in delivering the diagnosis of MSA in our inclusion criteria. In practice, however, delivering the diagnosis of MSA may not necessarily be the exclusive responsibility of experts in MSA practice. As such, the results of this survey may well reflect the status and experience of delivering MSA diagnosis in clinical practice throughout Japan.

## Conclusions

The findings presented herein revealed that a majority of neurologists perceived delivering the diagnosis of MSA as challenging. Most neurologists also perceived explaining the risk of sudden death, a unique feature of MSA, as difficult, which might be associated with the difficulty of breaking the diagnosis of MSA. Results from the quantitative and qualitative data revealed that empathic burden on neurologists caused by the progressive and incurable nature of MSA, the need to explain complex and important details, including the importance of the family decision-making process in life-prolonging treatment, difficulty in MSA diagnosis, and communication barriers posed by mental status and cognitive impairment of patients or their family members contributed to the difficulty in delivering diagnosis of MSA. Qualitative analysis also revealed that neurologists consider various factors, such as patient’s personality, mental state, and degree of acceptance and understanding, in explaining the risk of sudden death and adjust their manner of communication, such as limiting their communication on the risk of sudden death or avoiding the use of the term “sudden death” at the early stages of the disease. Although neurologists struggle with the need for various adjustments and empathic burden when delivering bad news on MSA, the current situation leaves room for improvement across multiple aspects, including the setting at which they deliver the diagnosis of MSA, the brief duration of consultation, obtaining the patient’s invitation by ascertaining how much the patient wants to know, and providing information about patient support group. Further research, including surveys from the patient’s perspective, and the development of guidelines for delivering the diagnosis of MSA are therefore needed.

### Supplementary Information

Supplementary Material 1. 

Supplementary Material 2. 

Supplementary Material 3. 

## Data Availability

The datasets used and/or analyzed during the current study available from the corresponding author on reasonable request.

## Abbreviations

MSA: Multiple system atrophy
ALS: Amyotrophic lateral sclerosis
